# Tuberculosis control at a South African correctional centre: Diagnosis, treatment and strain characterisation

**DOI:** 10.1371/journal.pone.0277459

**Published:** 2022-11-11

**Authors:** Kathleen Baird, Halima Said, Hendrik J. Koornhof, Adriano Gianmaria Duse

**Affiliations:** 1 Department of Clinical Microbiology and Infectious Diseases, School of Pathology of the University of Witwatersrand and National Health Laboratory Service, Johannesburg, South Africa; 2 Centre for Tuberculosis, National Institute for Communicable Diseases, Johannesburg, South Africa; University of Cape Town, SOUTH AFRICA

## Abstract

**Background:**

Correctional centres provide ideal conditions for tuberculosis (TB) transmission and disease progression. Despite the high TB incidence and incarceration rate in South Africa, data from South African correctional centres are scarce. Thus, the study evaluated TB diagnosis, treatment initiation and completion, and identified prevalent *Mycobacterium tuberculosis* strains among detainees entering a South African correctional centre.

**Methods:**

This study was a prospective observational study that enrolled participants between February and September 2017 from a correctional centre located in the Western Cape, South Africa. All adult male detainees who tested positive for TB during admission screening were eligible to participate in the study. Sputum samples from enrolled participants underwent smear microscopy and culture. Strain typing was performed on culture-positive samples. The time between specimen collection and diagnosis, the time between diagnosis and treatment initiation, and the proportion of detainees completing TB treatment at the correctional centre were calculated.

**Results:**

During the study period, 130 TB cases were detected through routine admission screening (126 male, 2 female, 2 juvenile). Out of the 126 eligible male detainees, 102 were enrolled in the study (81%, 102/126). All TB cases were detected within 30 hrs of admission screening. The majority (78%, 80/102) of participants started treatment within 48 hrs of TB diagnosis. However, only 8% (9/102) of participants completed treatment at the correction centre. Sputa from 90 of the 102 participants were available for smear and culture. There was a high smear positivity, with 49% (44/90) of isolates being smear positive. The Beijing family was the most frequent lineage (55.2%) in the study.

**Conclusion:**

The strengths of the current TB control efforts at the correctional centre include rapid detection of cases through admission screening and prompt treatment initiation. However, a high number of detainees exiting before treatment completion highlights the need to strengthen links between correctional TB services and community TB services to ensure detainees complete TB treatment after release and prevent TB transmission.

## Introduction

Detainees disproportionally come from lower socioeconomic groups. Thus, detainees are already at high-risk of TB infection and active TB disease when they enter the correctional system. The environmental factors that increase TB transmission and the health factors that increase the risk of being infected and accelerate the progression of active TB disproportionally affect people of lower socioeconomic status [1]. Poorer and marginalised people are more likely to face overcrowded and substandard living conditions [2]. There is a higher prevalence of HIV, malnutrition, and substance misuse among lower socioeconomic groups [3]. Thus, a large number of individuals at high-risk for TB are entering the correctional system–an environment that exacerbates TB transmission [4, 5]. This results in a high prevalence of active and latent TB in correctional centres compared with the general population. In sub-Saharan Africa, the prevalence of TB in correctional centres has been estimated to be 6 to 30 times higher than the prevalence of TB in the general population [6].

South Africa has one of the highest TB burdens (615 cases per 100,000 population) [7] and one of the highest incarceration rates (294 detainees per 100,000 population) [8] globally. The combination of a high TB incidence and a high number of people detained means that South African correctional centres may be a significant TB reservoir.

Effective TB control in correctional centres protects detainees, staff, and the community at large. Systematic screening, diagnosing TB quickly and accurately, and appropriate treatment are core components of the End TB strategy and form the basis by which TB prevention and control in correctional centres can be strengthened [5, 9–12]. South Africa has adopted policies that directly address TB in correctional centres. Guidelines established by the National Department of Health and the Department of Correctional Services require all detainees to be screened for TB upon admission, biannually and on release [13, 14]. When entering the correctional centre, all detainees are screened via a TB symptoms questionnaire [13]. The biannual mass screening includes clinical diagnosis through chest X-ray [13]. Detainees with suspected TB are tested according to the South African national TB algorithm, which utilises Xpert MTB/RIF assays as first-line testing [15]. Onsite Xpert MTB/RIF testing in correctional centres was implemented in 2014 [16].

However, Information regarding TB in South African correctional centres is limited. Data on TB diagnosis, TB treatment initiation and completion, and TB strains from South Africa’s correctional centres are important to fully understand the scope of the problem and identify potential gaps in TB infection control policies at the correctional centres. This study evaluated the current TB control programme at a South African correctional centre in Western Cape Province, by assessing TB diagnosis, treatment initiation and treatment completion, and evaluating *Mycobacterium tuberculosis* strains among detainees entering the correctional centre.

## Methods

### Study design

This study was a prospective observational study that enrolled participants over a seven-month period between 15 February 2017, and 15 September 2017, at a correctional centre located in the Western Cape of South Africa.

### Setting

The correction centre is a maximum security facility designed to hold around 4,000 detainees but is chronically overcrowded. In August 2016, overcrowding levels were over 300% [17]. Remand detainees constitute 66.8% of detainees housed at the correctional center [18]. The majority of detainees are male (98%) [8]. The correctional centre has an established TB screening and testing programme. Screening for TB is integrated into the admission intake process. On admission, all detainees are given a TB screening questionnaire and Xpert MTB/RIF is conducted on all presumptive TB cases. Xpert MTB/RIF testing is conducted on-site at the correctional centres. However, when the laboratory technician is on leave, sputum samples are sent to an off-site TB laboratory for Xpert MTB/RIF testing. One mass X-ray screening event occurred during the study period.

### Study population

The study population consisted of all adult male detainees at the correctional centre who were diagnosed with pulmonary TB through admission screening. The study population included both remand and sentenced detainees. The study inclusion criteria were > 18-years-old, male, detained at the correctional centre and diagnosed with pulmonary TB by either Xpert MTB/RIF assay or culture. Being < 18-years-old or female were exclusion criteria for this study.

Women made up only 2% of the correction population at the study site and were housed in a separate facility [8]. Thus, results obtained from women participants could not be combined with results from male participants. It was not feasible to analyse results from women participants separately as the women sample size would be too small to draw any significant conclusions.

### Informed consent and participant enrolment

The detainees who fulfilled the inclusion criteria were identified. The participant information sheet and the informed consent form were available in English, Afrikaans, and isiXhosa–the three official languages of the Western Cape [19]. Demographic data and sputum samples were collected only after voluntary informed consent was obtained and documented

Provisions were made for obtaining consent from prospective participants who were illiterate. If a prospective participant was illiterate, a witness selected by the prospective participant was required. A witness had to be present during the entire consent process. The witness had to be impartial and could neither be a member of the research team nor correctional centre staff. The witness had to be literate. The witness’ role was (1) to attest that the information was accurately explained to and understood by the prospective participant and (2) to attest that the consent was voluntary and freely given by the participants. For illiterate prospective participants, consent required the participant’s thumb print and the signature of the literate witness selected by the participant.

Prospective participants entered the study voluntarily and could withdraw at any time without having to give a reason and without any prejudice to them or their future care. Participating detainees and non-participating detainees received the same level of medical care and had the same access to correctional centre services. No benefits, compensation, or privileges were given for participation.

### Demographic and clinical information

The TB Register and clinical files were reviewed for the following information: i) Birth Date, ii) HIV status, iii) date and time sputum collected for first-line testing, iv) date and time of first-line Xpert MTB/RIF test, v) TB case category (new case/tretretment case), vi) treatment start date, and vii) treatment completion date. The detention status (remand or sentenced), the date participating detainees exited the correctional centre, and the reason for exiting were also recorded. Reasons for a detainee no longer being at the correctional centre included the following: i) not returned from court, ii) granted bail, iii) sentence ended, and iv) transferred to another correctional centre. For each participating detainee, the time from the screening sputum specimen collection to the time the Xpert MTB result was reported was calculated. The time it took for treatment to be initiated after a positive Xpert MTB result was calculated. The proportion of detainees exiting the correctional centre before completing the intensive phase and exiting before completing the continuation phase of TB treatment were calculated.

### Study sputum sample collection and processing

A sputum sample was collected from each enrolled detainee before the start of treatment and up to 72 hrs after the initiation of treatment. Sputum samples were specifically collected for this study and were additional to any routine sputum collections that occurred for the screening, diagnosis, and management of TB. OMNIgene-SPUTUM (DNA Genotek, Ottawa, Canada) was added at the correctional centre to allow ambient temperature transport of the sputum samples to the laboratory in Johannesburg. Smear microscopy (Ziehl-Neelsen stain) and culture on Löwenstein-Jensen media were performed. The total number of smear-positive cases among culture-positive cases was determined.

### Molecular analysis

*M*. *tuberculosis* positive cultures underwent strain typing using spoligotyping and 24-loci mycobacterial interspersed repetitive units-variable number tandem repeats (MIRU-VNTR). Analysis of the spoligotyping data was performed by assigning Spoligotype International Type (SIT) numbers and lineage designations using the SITVIT2 database (http://www.pasteur-guadeloupe.fr:8081/SITVIT2/) [20]. Spoligotype patterns were assigned an SIT if they shared identical spoligotype patterns with patterns present in the existing database. Spoligotypes that had no match with patterns in the SITVIT2 database represented unique patterns and were considered orphans. The MIRU-VNTRplus database (http://www.miru-vntrplus.org/) [21, 22] was used for the analysis of MURU-VNTR data.

### Statistical analysis

Chi-Square test was used to analyse binary variables. T-test was used to analyse continuous variables. A *p-value* < 0.05 was considered statistically significant.

### Ethical clearance

Ethical clearance was received from the University of the Witwatersrand Research Ethics Committee (Medical) (Certificate#: M140252) and the South African Department of Correctional Services Review Board.

## Results

During the study period, 22,346 detainees were admitted to the correctional centre of which 16,142 were screened for TB on admission (72%). All TB cases at the correctional centre during the study period were detected through admission screening. No detainees were diagnosed through self-presentation or through mass screening during the study period.

Admission screening resulted in 130 cases of pulmonary TB being detected (126 male, 2 female, 2 juvenile). Of the 126 adult male detainees at the correctional centre who were diagnosed with pulmonary TB during the study period (February 2017 to September2017), 102 were enrolled in the study (81%, 102/126). Six of the male detainees that were not enrolled in the study left the correctional centre before treatment iniation thus, there was no opportunity to obtain their consent.

All enrolled participants provided a sputum sample. However, 10 sputum samples leaked and had to be discarded. One sputum sample was lost in transit and another sputum sample was unsatisfactory. Attempts to recollect sputum samples from these 12 detainees were made but were unsuccessful. Thus, smear and culture were performed for 90 of the 102 enrolled detainees. Forty-nine percent (44/90) of samples were smear-positive. Seventy-four percent (67/90) of the samples were culture positive. The smear positivity among culture-positive isolates was 66% (44/67).

The demographic and clinical information of the participating detainees is presented in Table 1. The median age of the enrolled participants was 30 (IQR: 25–35). Seventy-five percent (76/102) of the participating detainees were HIV negative. A prior history of TB was reported by 35% (36/102) of detainees. All participating detainees were TB rifampicin-sensitive and followed the standard treatment regimen of an intensive phase lasting two months and a continuation phase lasting four months.

**Table 1:** Demographics and clinical information of participants.

		Enrolled		Sputum Provided			Culture-Positive	
		(n = 102)		(n = 90)			(n = 67)	
**Age**	Median	30		30			30	
	IQR	25–35		25–35			25–35	
**Detainee Status**	Remand	75	(74%)	64	(71%)		48	(72%)
	Sentenced	27	(26%)	26		(29%)	19	(28%)
**HIV Status**	Negative	76	(75%)	68		(76%)	52	(78%)
	Positive	26	(25%)	22		(24%)	15	(22%)
**TB History**	New Case	66	(65%)	57		(63%)	47	(70%)
	Retreatment	36	(35%)	33		(37%)	20	(30%)

IQR = interquartile range

There was no significant difference in age (p = 0.39), HIV status (p = 0.51), detainee status (remand or sentenced) (p = 0.13), and TB history (p = 0.43) between enrolled participants who had a sputa and enrolled participants without a sputa sample available for smear and culture. There was no significant difference in age (p = 0.36), HIV status (p = 0.43) and detainee status (remand or sentenced) (p = 0.84) between culture positive and culture-negative detainees. However, retreatment cases were more likely to have a negative culture result compared with new TB cases (p = 0.022).

### TB diagnosis and treatment initiation

All Xpert MTB/RIF results were reported within 30 hrs of specimen collection. The median turnaround time (TAT) from the time the sputum specimen was collected to the time the Xpert MTB/RIF result was reported was 7.13 hrs (IQR: 5:38–23.97). Seventy-Six percent (78/102) of samples were tested on-site while 24% (24/102) of samples were sent to an external NHLS laboratory. The TAT for onsite and offsite testing are presented in Table 2. On site testing had significantly shorter TAT then tests that were sent off site (*p* = 0.006).

**Table 2:** Time to diagnosis and time to treatment initiation.

	n	Hours to Diagnosis^+^		Hours to Treatment*	
		^Median [IQR]^		^Median [IQR]^	
**All participants**	102	7.13	[5.38–23.97]	24	[24–48]
**Xpert test conducted onsite**	78	5.85	[4.30–24.00]	24	[24–48]
**Xpert test conducted offsite**	24	15.98	[15.10–16.85]	48	[24–96]

^+^ calculated from the time of admission screening specimen collected to the time of Xpert MTB-positive result reported

* Calculated from date of positive Xpert MTB test to date of treatment initiation

The median time, from the time the Xpert MTB result was reported to the time treatment was initiated, was 24 hrs (IQR:24–48). The majority (78%,80/102) of participants started treatment within 48 hrs of receiving a positive Xpert MTB result. Within 72 hrs of receiving a positive Xpert MTB result, 87% (89/102) of participants had started treatment. Out of the nine cases that started treatment 72 hrs after the positive Xpert MTB result, eight were cases where the Xpert MTB test was conducted on Friday and treatment initiated on Monday.

For seven cases, there was a substantial delay in treatment initiation (5–9 days after positive Xpert MTB result). Six of the cases that had a substantial delay in treatment initiation were samples that were sent to an external NHLS Laboratory for Xpert MTB testing and were not tested onsite at the correctional site. For six participants information on treatment start date was not available.

### Treatment completion

**Fig 1:**
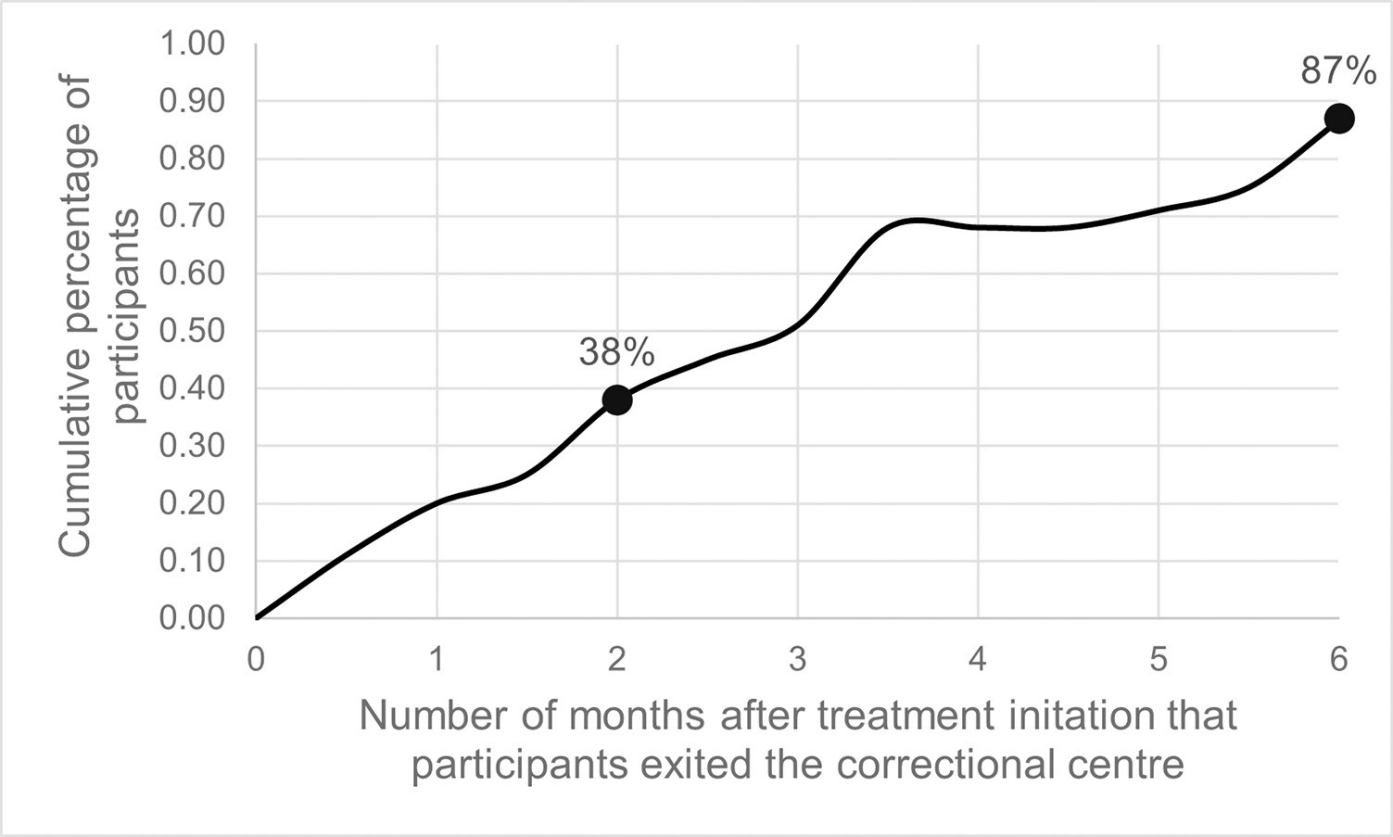
The percentage of detainees exiting the correctional centre before treatment completion. All detainees were rifampicin-susceptible and followed a treatment regime consisting of a 2-month intensive phase followed by a 4-month continuation phase. 36% of detainees exited the correctional centre before the completion of the intensive phase of treatment. 87% were outside the correctional centre when treatment should have been completed.

Participants were highly mobile (Fig 1). Thepercentage of participants who returned to their communities before completing the intensive phase of treatment (i.e. detainees exiting within two months of treatment initiation) was 38% (39/102). Not returning from the court was the most common reason for not completing the intensive phase of treatment at the correctional centre (59%, 23/39). Granted bail was the second most common reason for leaving the correctional centre before completion of the intensive phase of treatment (15%, 6/39).

The percentage of participants who completed the intensive phase of treatment at the correctional centre but did not complete the continuation phase of treatment before returning to their communities (i.e.detainees exiting the correctional centre was between two and six months after treatment initiation) was 46% (47/102). Only 8% (9/102) of participants remained at the correction centre for longer than six months and completed TB treatment (completed both the intensive and continuation phase) at the correctional centre (Fig 1). The exact exit date and treatment status at the time of exit were unavailable for 4% (4/102) of study participants.

### Molecular analysis

All 67 *M*. *tuberculosis-*positive cultures underwent molecular typing. Comparison of spoligotyping results with the SIVIT2 database showed that 93% (62/67) of isolates belonged to 15 previously described Spoligotype International Types (SITs). Spoligotype patterns for five isolates were not found in the SIVIT2 database and these isolates were considered orphans. The most dominant *M*. *tuberculosis* lineage was Beijing and was represented by 55% (37/67) of isolates. Euro-American was the second most prominent lineage with 34% (23/67) isolates. Two isolates belonged to SITs that were not associated with any lineage (SIT 4 and SIT 46).

## Discussion

There have been very few studies conducted in sub-Saharan Africa focused on active TB case finding interventions in correctional centre [23]. Our findings showed smear positivity among detainees entering the correction to be high with 66% of culture-positive TB cases being smear-positive. TB cases were being detected within 30 hrs of admission screening and the majority of participants started treatment within 48 hrs of TB diagnosis. However, only 8% participants completed treatment at the correction centre.

Individuals with a high bacterial load are highly infectious and remain infectious for longer [24]. As a positive smear indicates a high bacterial load, smear-positivity is commonly used as an indicator of infectiousness. Thus, the high number of smear-positive TB cases observed among detainees entering the correctional centre underscores the need for strong TB control measures at the correctional centre. As the most infectious pulmonary TB patients are those with smear-positive/culture-positive pulmonary TB, the high percentage of smear-positivity among culture-positive samples (67%) observed further confirms that individuals are entering the correctional centre with highly infectious TB.

Discordance between Xpert and culture results has been reported in other studies [25, 26]. In our study, retreatment cases were more likely to have a negative culture. Determining whether these retreatment cases with negative culture had a false-positive or true-positive Xpert MTB result was beyond the scope of this study. Meta-analyses of Xpert studies show that Xpert MTB has a pooled sensitivity of 61.8%–87% and specificity of 98%–98.8% for pulmonary TB [27, 28]. The Xpert MTB/RIF assay detects DNA of both viable and non-viable organisms, and it is unclear how long after treatment completion TB patients remain Xpert MTB positive. One study found that 27% of patients were still Xpert MTB positive 26 weeks after treatment initiation [29]. Additionally, prior exposure to anti-TB drugs reduces the density of tubercle bacillus and weaken surviving bacteria in the sputum [30]. Thus, retreatment cases having a negative culture were an expected result.

The prevalence of TB among HIV-positive detainee is high worldwide [31]. The intergration of TB and HIV services is an essential component of both the global response and South Africa’s response to the TB/HIV dual epidemic [32, 33]. However, the majority of participants in this study were HIV negative. This is consistant with the South African national TB prevalence survey which found a higher prevalence of undiagnosed TB among HIV-negative individuals [25]. Thus, efforts to stengthen TB control measures need to ensure sustained engagement in care of released detainees who are TB-positive but HIV-negative.

In the South African national TB prevalence survey, young males had the highest number of undiagnosed or unreported TB cases; and 79% of individuals surveyed between the age of 25–34 did not seek care [25]. There is an overrepresatation of young males entering correctional centres. Thus, correctional TB programmes provides an opportunity to reach young males, who typically have lower engagement with community health services and where TB is underdiagnosed and underreported. The strengths of the current Department of Correctional Services TB control programme include rapid detection of TB cases through admissions screening and prompt treatment initiation (78% (80/102) of participants started treatment within 48 hrs of diagnosis). For samples sent off site there was no delay between sample collection and testing. But off-site testing resulted in delays between the time Xpert MTB results were first reported and treatment initiation. Streamlining the process of communicating results from the external TB laboratory back to the correctional centre would help minimise delays in treatment and strengthen the current control programme.

Rapid diagnosis and treatment initiation are only two of the key components of effective TB control. Successful treatment completion is another key component of TB infection control. Due to a high level of mobility, most detainees (87%) were outside the correctional centre when treatment should have been completed. It is possible that some detainees in this study sought care and continued their treatment once released, but confirming this was beyond the scope of this study. However, low rates of TB treatment completion among released detainees are well documented. A study at a correctional centre in the North West Province of South Africa found that 59% of study participants were outside the correctional centre (released or transferred) when treatment should have been completed, and less than 50% of participants in that study completed treatment or had documented cure [34]. A study conducted in Uganda found that 81% of the detainees released were lost to follow-up [35]. Being released before treatment completion has implications for both individuals and the community at large. Individuals who do not complete treatment have an increased risk of relapse, drug resistance and mortality [36–39]. Individuals who do not complete treatment also have prolonged infectiousness [40, 41]. Thus, released detainees who have not completed TB treatment raise the overall risk of TB in surrounding communities. A study conducted in South Africa found communitiess that contained at least one correctional centre had approximately one-third more detected cases of TB than communities that did not contain a correctional centre [42]. A low level of treatment completion will impede control efforts at the correctional centre and drive transmission within the community.

The high turnover of detainees observed in our study was partly a result of efforts by the Department of Correctional Services to reduce overcrowding at the correctional centre. By May 2017, overcrowding at the correctional centre was reduced to 149% [43]. However, with the decrease in overcrowding, the correctional centre is now primarily accommodating remand detainees. The increase in remand detainees and the resulting increase in turnover at the correctional centre may further drive TB transmission within the surrounding communities. A study in Zambia observed that correctional centers with a higher proportion of remand detainees, subsuquently had a higher rates of release and a higher number of TB patients lost to follow-up [44].

Hippner et al. [45] showed that improving treatment completion was a cost effective method for strengthening TB control in the Western Cape. Avoiding the release of detainees undergoing TB treatment would make achieving treatment completion more straightforward. However, this is neither a practical nor ethical solution as it infringes on the rights of detainees and hinders correctional centre management. Thus, improvements must be made in the continuation of care for released detainees.

Currently, the responsibility is placed on the released detainee to ensure that their treatment is continued once released. The responsibility of continuum of care should not be placed on TB patients but on TB programmes. Both the correctional and community TB programmes need to be held accountable for treatment completion of released detainees. Establishing accountability will motivate both the correctional centre and community TB programmes to communicate and collaborate to ensure a continuum of care of released detainees.

The following recommendations should be addressed simultaneously to improve treatment completion and decrease the TB burden: i) Create accountability. Both the correctional and community TB programmes need to be held accountable for treatment completion of released detainees; ii) Establish comprehensive discharge plans for detainees on TB treatment. As not returning from court was the main reason participants did not complete the intensive phase of treatment at the correctional centre, discharge plans need to be in place before detainees head to court.

There is a need to stregnth correctional health information systems to enable better linkage and tracking between correctional centres and community health services [46]. The National Strategic Plan for HIV, STIs, and TB 2017–2022 has addressed the need for an electronic national health information system to track patients through the continuum of care [33]. However, an intergrated national health information system will only be effective if information is updated into the system accurately and in a timely manner.

The molecular findings of this study further illustrate the interconnectedness between TB cases at the correctional centre and TB in the broader community. The two most prevalent lineages observed in this study were Beijing and Euro-American. This is consistent with other investigations that have shown that the majority of isolates in the Western Cape belong to Beijing and Euro-American lineages [47, 48]. Molecular evidence from Brazil supports the linkage between TB cases in correctional centre and in the surrounding communities [49, 50]. Sacchi et al. found 54% of Mycobacterium tuberculosis strains in the community were related to strains from detainees suggesting spread between the two populations [49]. In the study by Walter et al. [50] the majority of genomic clusters included both individuals with a recent history of incarceration and individuals with no incarceration history. The study concluded a minimum of 16.8% of infections among never incarcerated invididuals were directly attributable to spillover from TB cases at the correctional centre [50].

One limitation of the study was that it did not include all TB cases detected during the study period. This study was prospective study and consent was required to access clinical and correctional information. While a retrospective data review was considered, it was not feasible because of scant and incomplete records. The prospective nature of the study allowed us to verify the accuracy of data obtained from the clinical and correctional files and actively resolve discrepancies and complete missing data. While this design meant we did not have access to all TB cases during the study, this approach improved the robustness of the data obtained and our confidence in the findings. This approach also meant that the findings better reflect the present situation at the centre.

Another limitation of the study was that Xpert MTB/RIF, smear microscopy and culture were not performed on all newly admitted detainees, regardless of symptoms. The South African national TB prevalence survey found that the majority of bacteriologically confirmed TB cases had no reported TB symptoms [24]. Sub-clinical cases would not be detected by the screening symptom questionaire. A study conducted in South African correctional centres found digital chest X-ray identified two times more patients with undiagnosed TB than when sympton questionaire was used alone [51]. By only focusing on symptomatic cases in this study, there is a risk of bias and the results may not accurately represent TB infections entering the correctional centres. However, by focusing on symptomatic individuals, this study looked at the group of detainees who were most infectious. Symptomatic individuals are an important subgroup as they are the main drivers of transmission. Thus, focusing on limited TB resources, especially in areas with high prevalence, on identifying symptomatic individuals and ensuring they compete TB treatment will have a significant impact on TB control efforts.

The last limitation of this study was that it was conducted in a single correctional centre. The correctional centre was selected because it was the correctional centre in the Western Cape that reported the highest TB burden and it had an established TB screening programme. The recommendations presented address the unique situation of the study site. Thus, caution is advised when generalizing the findings. The recommendations presented may not be appropriate or practical for other correctional centres. However, other correctional centres can use the findings and recommendations presented as a starting point that can be adapted to fit their specific environment and needs. Although limited in scale and scope, our research is an important step in establishing an overall picture of TB in South African correctional centres. It adds to the current understanding of the TB in South African correctional centres and identifies ways to strengthen current TB control measures.

## Conclusion

TB in the correctional centre and TB in the surrounding communities are intrinsically linked. Detainees were entering the correctional centre from the community with undiagnosed TB. TB admission screening at the correctional centres provides an opportunity to identify TB cases that have gone undetected by community TB control activities. However, successful treatment completion is necessary for admission screening to have an impact on TB transmission. The high number of smear-positive cases and the high number of detainees being released before treatment completion mean that detainees are reentering the community with potentially infectious TB. The ability to ensure completion of treatment is necessary for the correctional TB programme to be effective. Thus, for TB efforts at the correctional centre to be successful, they need to collaborate and strengthen ties with community TB programmes. Improving treatment completion among released detainess will not only result in improved individual health outcomes but have a positive impact on the broader community.
